# MARINE-EXPRESS: taking advantage of high throughput cloning and expression strategies for the post-genomic analysis of marine organisms

**DOI:** 10.1186/1475-2859-9-45

**Published:** 2010-06-14

**Authors:** Agnès Groisillier, Cécile Hervé, Alexandra Jeudy, Etienne Rebuffet, Pierre F Pluchon, Yann Chevolot, Didier Flament, Claire Geslin, Isabel M Morgado, Déborah Power, Margherita Branno, Hervé Moreau, Gurvan Michel, Catherine Boyen, Mirjam Czjzek

**Affiliations:** 1 UPMC Univ Paris 6, UMR 7139 Végétaux marins et Biomolécules, LIA DIAMS, Station Biologique, F 29682, Roscoff, France; 2 CNRS, UMR 7139 Végétaux marins et Biomolécules, LIA DIAMS, Station Biologique, F 29682, Roscoff, France; 3 Ifremer-CNRS-UBO, UMR6197, Microbiology of Extreme Environments, Technopole Brest-Iroise, BP70, 29280 Plouzané, France; 4 Institut des nanotechnologies de Lyon (INL), UMR5270, Ecole Centrale de Lyon Bât. F 7, 36 Av Guy de Collongue, 69134 Ecully Cedex, France; 5 Centro de Ciências do Mar, Universidade do Algarve, Comparative and molecular Endocrinology, 8005-139 Faro, Portugal; 6 Cellular and Developmental Biology, Stazione Zoologica A. Dohrn, Villa Comunale, 80121 Napoli, Italia; 7 Observatoire de Banyuls sur mer, Models in Cellular and Evolutive Biology, UMR7628, CNRS-UPMC. Banyuls sur mer, France

## Abstract

**Background:**

The production of stable and soluble proteins is one of the most important steps prior to structural and functional studies of biological importance. We investigated the parallel production in a medium throughput strategy of genes coding for proteins from various marine organisms, using protocols that involved recombinatorial cloning, protein expression screening and batch purification. This strategy was applied in order to respond to the need for post-genomic validation of the recent success of a large number of marine genomic projects. Indeed, the upcoming challenge is to go beyond the bioinformatic data, since the bias introduced through the genomes of the so called model organisms leads to numerous proteins of unknown function in the still unexplored world of the oceanic organisms.

**Results:**

We present here the results of expression tests for 192 targets using a 96-well plate format. Genes were PCR amplified and cloned in parallel into expression vectors pFO4 and pGEX-4T-1, in order to express proteins N-terminally fused to a six-histidine-tag and to a GST-tag, respectively. Small-scale expression and purification permitted isolation of 84 soluble proteins and 34 insoluble proteins, which could also be used in refolding assays. Selected examples of proteins expressed and purified to a larger scale are presented.

**Conclusions:**

The objective of this program was to get around the bottlenecks of soluble, active protein expression and crystallization for post-genomic validation of a number of proteins that come from various marine organisms. Multiplying the constructions, vectors and targets treated in parallel is important for the success of a medium throughput strategy and considerably increases the chances to get rapid access to pure and soluble protein samples, needed for the subsequent biochemical characterizations. Our set up of a medium throughput strategy applied to genes from marine organisms had a mean success rate of 44% soluble protein expression from marine bacteria, archaea as well as eukaryotic organisms. This success rate compares favorably with other protein screening projects, particularly for eukaryotic proteins. Several purified targets have already formed the base for experiments aimed at post-genomic validation.

## Background

The marine environment is highly complex and contains the vast majority of known and unknown biodiversity. It is also the last frontier to understand the control of the global climate and hides a wealth of biological resources still to be tapped for food, health and energy. Up until very recently, few genomic data were available for oceanic organisms, but this panorama is rapidly changing with a number of genomic projects now underway, which focus on marine organisms, ranging from microbes [1,2] to multicellular eukaryotes including vertebrates or macro-algae [3], as well as the generation of resources and access to genomes or EST libraries for various eukaryotic systems [4-7]. The wealth of sequence data arising from these projects, means that researchers are confronted with a huge number of putative genes, the function of which are, at best, so far only deduced from sequence comparisons (automatic annotation). The pressing question is how to analyze the genomic data with respect to original biological processes in diverse marine organisms (i.e. their development or stress response), their importance in adaptation to the particular habitat and how to identify new enzymes and/or metabolites of biotechnological interest. Thus, the availability of complete genome data has resulted in the development of transcriptomic and proteomic methods that can be used to study regulatory networks and interactions of thousands of genes in parallel, allowing an efficient global analysis of genomic information. However, there are a number of clear drawbacks with these methods in so far that they are strongly dependent on the quality of the genome annotation, which at present assumes conserved functions across often widely distant taxa. Furthermore, these techniques give at most only an indication of the regulation/metabolic pathway the corresponding gene product belongs to, and little or no information on the precise biochemical function of unknown genes.

To understand the precise biological function of a single gene, the biochemical and physiological characterization of its product is essential and this is often greatly aided by the availability of 3-D structural information. Although the 3D structure does not always reveal the natural substrate, it has been shown repeatedly that it helps at least find the class of compounds among which the substrate will be found [8,9]. Several bottlenecks exist in the analysis of individual proteins; generally the techniques utilized require systems for the efficient over-expression of the target gene in order to produce sufficient recombinant protein. Furthermore, to constitute an assessment for potential biotechnological applications of the discovered proteins/enzymes, the effective recombinant expression of biologically active proteins is essential. When aiming at the 3D-structure of the protein of interest, a second bottleneck is encountered at the step of crystallization of these proteins. Recent developments in the field of structural genomics have demonstrated that medium/high throughput strategies are most adapted to the production of large numbers of soluble and active gene products and/or protein crystals at a time [10,11], since they allow simultaneous testing of numerous conditions with an optimized effort.

In this study, our objective was to set up and apply a medium throughput strategy for the production, expression and, at least for some candidates, the crystallization of selected proteins from marine organisms. With this approach, we targeted proteins from various marine organisms including archaea, bacteria and eukaryotes but also proteins from different families of interest as for example enzymes involved in the metabolism of carbohydrates, stress related proteins or cellular division proteins (Table 1). On the basis of its ease of genetic manipulation, its rapid growth rate, low cost and robustness *Escherichia coli *still remains the most popular expression system [12]. Moreover, the results of high-throughput programs (such as those funded under the NIH Protein structure Initiative (PSI) [13] available on the site PSI/targetDB http://targetdb.pdb.org/ show that in term of full-length proteins, up to 50% of proteins from the Eubacteria or Archaea and 10% of proteins from Eukarya can be expressed in *E. coli *in soluble form [14]. We have therefore chosen this expression system for our purpose and report the results of the parallel expression strategy in *E. coli *192 targeted genes, in two compatible expression vectors, using a 96-well plate format. A selection of individual targets that were successfully overexpressed in this program, were subsequently further characterized in order to determine their functional partners, cellular localization, 3D structure and/or biochemical activity. As a conclusion, three selected examples of these studies are briefly discussed.

**Table 1:** Availability of genomic data to the Marine Express partners.

Organism	Taxonomy	Gene families of interest	State of genomic data	Partner
***Pyrococcus abyssi***	Euryarchaeota	Proteins from the DNA replication system	Genome published [1]	IFREMER Brest, France

***Zobellia galactanivorans***	Flavobacteria	Polysacchride metabolism, sulfatases	Genome complete	SBR Roscoff, France

***Ectocarpus siliculosus***	Brown alga	Stress related genes, carbohydrate active enzymes	14000 ESTs, Genome complete	SBR Roscoff, France

***Ostreococcus tauri***	Green microalga	Biological clock	Genome published [4]	Arago Banyuls, France

***Ciona intestinalis***	Urochordate	Hox-genes, Ci-msx, Ci-RX	Genome published [5]	SZ A. Dohrn, Napoli, Italia

***Sparus aurata***	Teleost fish	Hormones, calcium and musculo-skeletal development, stress related	30000 ESTs and 20 full length cDNAs	CCMAR Faro, Protugal

***Archaeal virus (PAV1)***	Not yet classified	Any	Genome published [28]	UBO, LM2E, Brest, France

## Results

### Selection and bioinformatics analysis of the target genes

A subset of 200 target genes was selected by the different partners involved in the program to evaluate the potential for heterologous expression of marine organism genes in *E. coli*. The gene families of interest, defined by the partners and organized according to the organism of origin and from three domains of life, are shown in Table 2. The sequences of the genes and their corresponding predicted proteins were analyzed by bioinformatic tools to assess their suitability for expression in our bacterial medium throughput strategy. The nucleotide-sequences were screened for the absence of the selected restriction sites for the gene cloning strategy. Where possible, the potential signal peptides and transmembrane domains were removed in order to express soluble proteins. These were therefore identified using SignalP and TMHMM servers, respectively [15,16]. Another important feature that was analyzed in this step was the protein modularity. It is often assumed that single domains of proteins contain important biological functions and are more likely to be successfully expressed than the large, multi-modular proteins. Moreover, the modularity of a protein is usually an obstacle to its crystallization. Consequently, targeted proteins were, where possible, cloned as full length proteins, but also in the form of their individual modules. The modular architecture of each target was examined using Blast queries against UniProt database, as well as domain searches with the InterPro server [17]. The crucial choices of the N- and C-terminal boundaries of each module were refined using Hydrophobic Cluster Analysis (HCA) [18]. From 200 starting sequences, eighteen were eliminated after restriction site analysis and eight because of the presence of transmembrane zones throughout the protein sequences.. Finally, 192 sequences encoding proteins ranging from 7 to 140 kDa were analyzed and classified according to different restriction site strategies as follows: *Bam*HI/*Eco*RI, *Bam*HI/*Mfe*I, *Bgl*II/*Eco*RI and *Bgl*II/*Mfe*I.

**Table 2:** Comparison of obtained results covering three domains of living organisms

	Eukarya		Bacteria		Archaea		Total		
	**pFO4**	**pGEX**	**pFO4**	**pGEX**	**pFO4**	**pGEX**	**pFO4**	**pGEX**	**combined^c^**

number of soluble proteins	14	13	13	25	18	26	45	64	84
number of insoluble proteins	10	19	4	5	12	24	26	48	34
no expression^a^	42	37	11	2	31	11	84	50	52
no clone^b^	8	5	4	0	9	12	37	30	22

percentage of soluble proteins	7%	7%	7%	13%	9%	14%	23%	33%	44%
percentage of soluble + insoluble proteins	13,00%	17%	9%	16%	16%	26%	37%	58%	61%

^a ^number of targets for which no expected bands were visualized on SDS-PAGE gels

^b ^number of target genes did not clone in expression vectors

^c ^number of different soluble proteins obtained in pGEX or in pFO4 vectors

### Amplification and cloning of DNA fragments

To optimize the amplification steps through PCR, specific primers were designed which had the same theoretical T_m _value for all targets.

**Figure 1:**
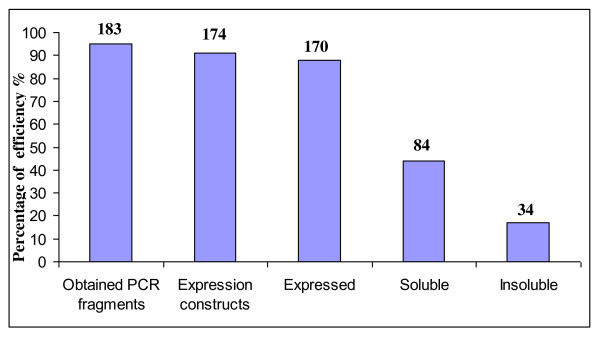
**Comparison of protein expression screening success**. The percentage of success for each step in the protein production pipeline is shown. The number of targets represented at each stage is written above the bars.

The starting genetic material was genomic DNA for prokaryotes and full length cDNA for eukaryotic organisms. Therefore, in parallel to the bioinformatic analysis, an important step for eukaryotic proteins was to retrieve full length cDNAs from the genetic material. This step was performed separately by each partner by 5'RACE PCR on available cDNA libraries. Selected target genes were amplified by PCR using a general set of gene-specific primers (cf Methods). Even non-amplified targets were included in subsequent steps and permitted recovery of two genes in the cloning step. Overall, 183 out of 192 PCR fragments were obtained (Figure 1). These recovered, non-amplified genes corresponded to cDNA (obtained in weak concentration) or genomic DNA with a size greater than 3500 base pairs.

In the present study, two expression vectors were used to test recombinant protein expression and solubility. One vector (pFO4) contains a His_6_-tag at the N-terminal for affinity purification; the second (pGEX-4T-1) allows the production of a fusion protein with an N-terminal glutathion-S-transferase (GST). This parallel PCR cloning procedure could easily be performed in 96-well format. The approach relies on the use of a single PCR product for each gene that is compatible for ligation to both the expression vectors, pGEX-4T-1 and pFO4. Since the expression vectors were digested with *Bam*HI and *Eco*RI, the upstream and downstream PCR primers introduced *Bam*HI (or its isocaudomer *Bgl*II) and *Eco*RI (or its isocaudomer *Mfe*I) restriction sites, respectively, upon PCR amplification. After transformation in *E. coli *DH5α strains, the plasmids were validated by PCR screening of colonies using primers specific for the expression vectors and flanking the cloning sites. In this way, we obtained 174 cloned target genes (Figure 1). The efficiency for direct cloning of these target genes from PCR products was 95% (174 out of 183).

### Expression and purification of recombinant proteins in small-scale experiments

The validated plasmids were used to transform appropriate *E. coli *expression strains (Table 3). Fusion proteins in pFO4 vector are under-control of a T7 promoter and in *E. coli *cells containing a chromosomally located defective prophage DE3 must be used for transformation. For Seventy-seven genes that were cloned from archaea Rosetta or Rosetta (DE3) strains were transformed, which compensate for a number of rare codons in *E. coli*. For cloned genes that contain several cysteines in their sequences Origami or Origami (DE3) strains (14 genes) were transformed. Indeed, these cells carry mutations for both the thioredoxin reductase (*trxB*) and glutathione reductase (*gor*) genes, mutations which greatly enhance disulfide bond formation in the cytoplasm. For all other recombinant vectors BL21 or BL21 (DE3) strains were used to be transformed.

For 167 out of 183 cloned constructs that contained inserts of the expected size small-scale experiments for soluble protein expression were screened. A key step in the automation of the small-scale experimental setup is the development of auto-induction media (ZYP5052 medium used in the present study), which contain differentially metabolized carbon sources that promote growth to relatively high cell densities and then auto-induce by the utilization of lactose. These media remove the need to monitor cell densities or to add an inducer such as IPTG in T7-based expression systems [19]. To further optimize soluble protein expression, we used a culture temperature of 20°C for 3 days, since it has been established by the *Structural Proteomics In Europe *consortium [20] and others that lower temperatures tend to be more effective [21]. Moreover, in the present study 24-well plates instead of the usual 96-well plates [14] were used to obtain better aeration of the cells [22]. The final OD_600 _values that were reached in the small-scale cultivation vessels ranged between 10 and 16.

**Figure 2:**
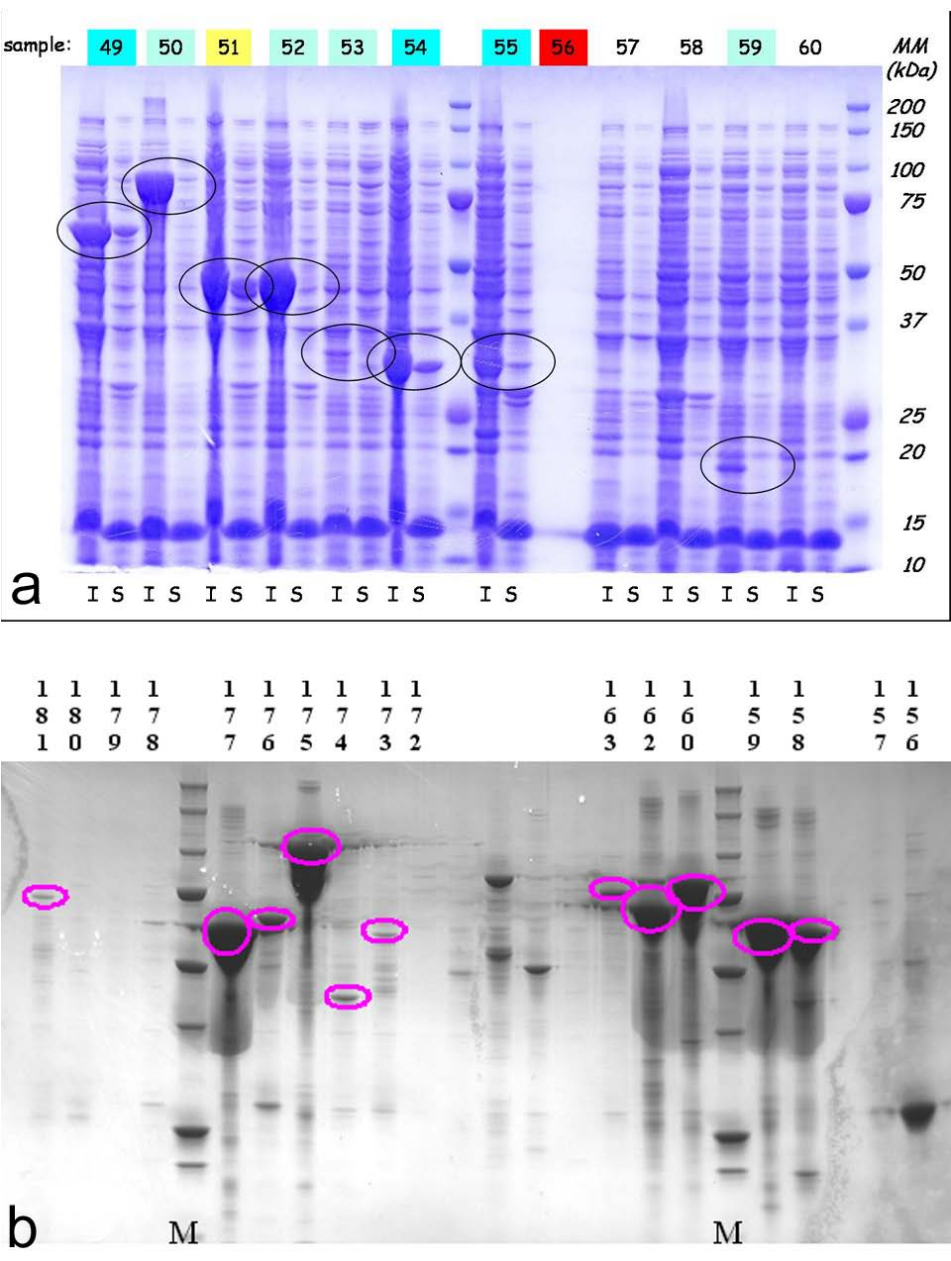
**Assessing soluble and insoluble protein expression levels in the medium throughput strategy**. **a**. SDS-PAGE of Insoluble (I) and soluble (S) crude cellular extracts of the *E. coli *expression cultures. Targets 50 to 60 are shown. Black circles surround bands of correct size for a given target. **b**. Western-blot using anti-His antibody of selected targets highlighted after purification of soluble cell lysis extracts on mini-affinity (His) columns. Selected targets between 156 and 181 are shown. Pink circles highlight bands of correct size for a given target.

The expression and the solubility of the recombinant proteins were tested by SDS-PAGE. Only for few target clones it was possible to visually detect the protein band corresponding to the fusion protein directly on SDS-PAGE gels, as illustrated for some examples in Figure 2a. Consequently, to validate the exact number of clones that expressed proteins in the soluble fractions, we used Microspin GST/HIS mini-columns (GE Healthcare Life Science, USA) to perform a mini-purification screen. Indeed, these mini-columns are used to purify a GST fusion protein or a HIS fusion protein by affinity chromatography. After incubation and washing, bound His-tagged or GST-tagged proteins were eluted with elution buffer containing imidazole or reducing-glutathion, respectively. The elution results were again analyzed on SDS-PAGE gels (Figure 2b). This evaluation method was much more sensitive and revealed that 118 proteins out of the 174 constructs (68%) were expressed, while 84 of these 118 (71%) were found to be soluble (Table 2 and Additional file 1, Table S1).

### Scale-up expression and purification of targets from *Pyrococcus abyssi*

**Figure 3:**
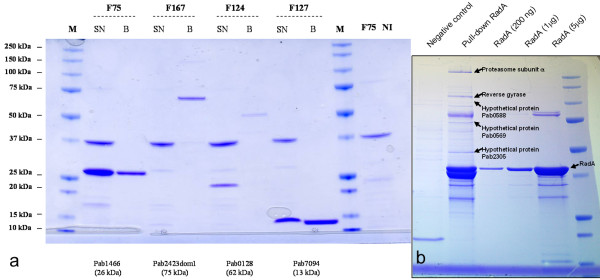
**Up-scale and purification of targets from P. abyssi**. **a**. SDS-PAGE gel of up-scaled and purified HIS-tagged targets F75, F124, F127 and F167 before and after final polishing on Co^2+ ^specific beads (SN = Supernatant of cell culture; B = after incubation with Dynabeads TALON; M = protein markers; F75 = test line; NI = F75 without induction). **b**. 12% SDS-PAGE analysis of a *P. abyssi *RadA (PAB0164, target 92 round R1) pull-down experiment. Proteins identified using an Ultraflex MALDI-TOF/TOF instrument (Bruker Daltonics) are indicated with arrows.

A relative high success rate (45% of solubly expressed targets) was obtained for targets originating from the hyperthermophilic archae *Pyrococcus abyssi*. A total of 37 out of 77 expressed proteins were expressed soluble and easily purified in the small scale set up. To pursue the biochemical characterization of these proteins, to date the purification of 33 proteins has been performed at a larger scale. The up-scaling was performed using culture volumes of 200 ml and based on the protocols of the medium throughput strategy. Independent of size or protein family, reasonable yields in the range of several mg of pure protein were obtained for the scaled-up targets, as exemplified by four targets in Figure 3a. One of the aims of the follow-up study based on the genome data of *P. abyssi *is to identify novel partners in the archaeal DNA repair/replication system. The purified and tagged proteins are therefore subsequently used in pull-down/MS experiments as previously described [23]. One example of a successful pull-down experiment is illustrated in Figure 3b. The up-scaled, produced and purified target 92 (PAb0164, see Additional file 1, Table S1) was immobilized on the column. Interacting proteins were co-purified and subsequently identified using an Ultraflex MALDI-TOF/TOF instrument (Bruker Daltonics).

### Up-scale, purification and crystallization of R-Z3597 from *Zobellia galactanivorans*

**Figure 4:**
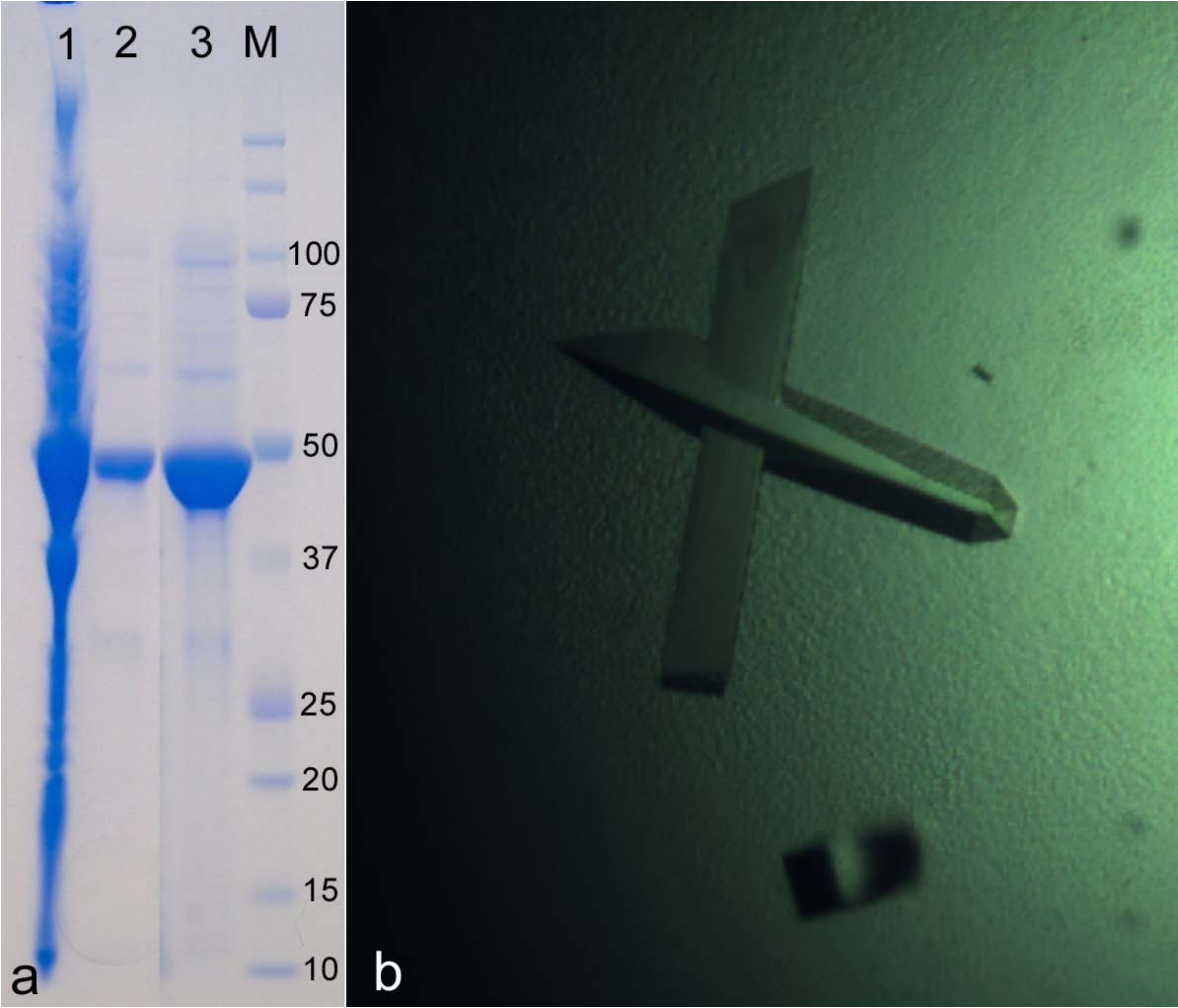
**Up-scale purification and crystallization of R-Z3597 from *Zobellia galactanivorans***. **a**. SDS-PAGE gel of up-scaled and purified His-tagged target Zg3597. Lane 1, soluble extract; lane 2, purified R-Z3597 by affinity chromatography using a Ni^2+^-charged resin; lane 3, R-Z3597 purified by size exclusion gel chromatography; M, standard molecular weight markers. **b**. Orthorhombic crystals of Zg3597 (0.1 × 0.1 × 0.5 μm^3^)

The genome of *Z. galactanivorans *contains approximately 50% of 'hypothetical proteins', as identified by bioinformatic analysis (Barbeyron et al., in preparation). A selection of these proteins, chosen because of very distant similarity to glycoside hydrolases or because they were collinear and found in close genomic context to other glycoside hydrolases, were targets for the heterologous expression, to produce sufficient pure protein samples for a complete structural and biochemical characterization. In particular, R-Z3597 (target 43, Additional file 1, Table S1) was successfully over expressed in soluble form and in high yield. The scale-up of expression was performed using the protocol established in the medium throughput trial, but for 500 ml culture medium. The final yield after two steps of purification (Nickel affinity and size exclusion) was 15 mg (Figure 4a). A solubility screen, following the strategy of Collins et al. [24] indicated that the best results were obtained with a buffer composed of 50 mM CHES pH 9.5, 500 mM NaCl and 100 mM imidazole. The protein was concentrated in this buffer to a final concentration of 12 mg/ml. Crystallisation conditions were screened using three different commercial kits (PEGI, PACT and JCSG+). Orthorhombic single crystals grew in the optimized condition, containing 20% (w/v) PEG 3350 and 200 mM calcium acetate, within one or two weeks at 292 K (Figure 4b). The further biochemical characterization, especially the screening for activity on various marine oligo- and polysaccharides is currently in progress.

### Scale-up, purification and crystallization of Staniocalcin1A from *Sparus aurata*

**Figure 5:**
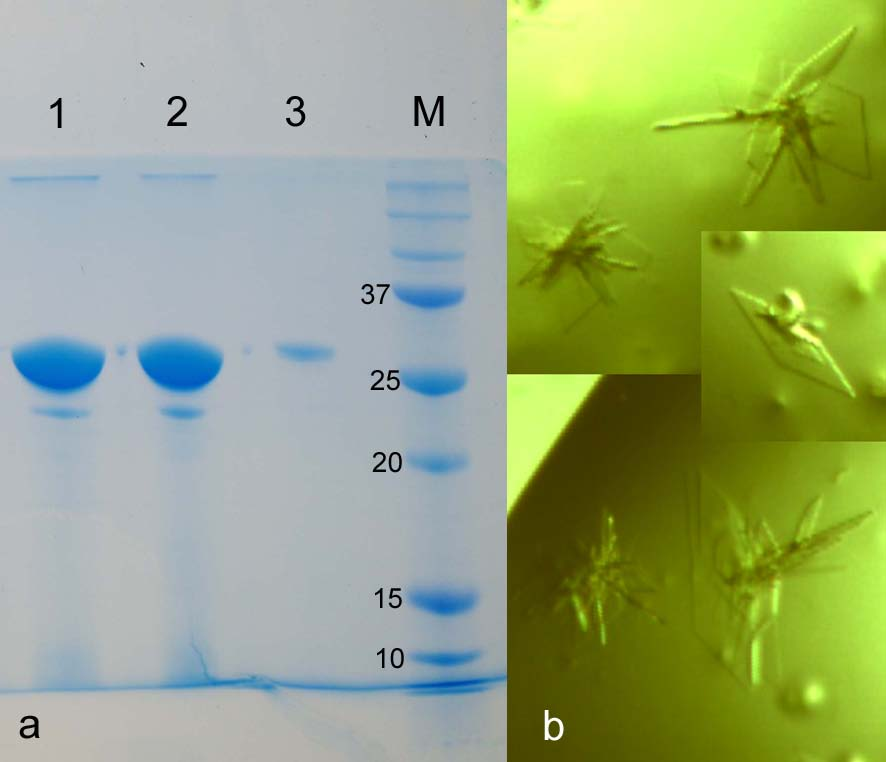
**Up-scale purification and crystallization of Stanniocalcin 1 of *Sparus aurata***. **a**. SDS-PAGE gel of up-scaled and purified His-tagged target STCA1. **b**. First plate-like crystals of STCA1 (approximate size of 0.05 × 0.05 × 0.008 μm^3^).

Functional characterization of proteins from *Sparus aurata *involved in handling and regulation of calcium: several categories of fish cDNA were provided to the flagship project MARINE-EXPRESS and included hormones, hormone responsive transcription factors, chaperones and matrix proteins involved in hormone driven calcium turnover. Target constructs used for further studies included sea bream and *Tetraodon nigroviridis *Stanniocalcins. In particular the expression of stanniocalcin1 (STCA1) from *Tetraodon nigroviridis *was scaled-up following the protocol established in the medium throughput trial, but for 500 ml ZYP5052 culture medium. The final yield after two steps of purification (Nickel affinity and size exclusion) was about 12 mg (Figure 5a).

Crystallisation conditions were screened using three variants of commercial kits (PEGI, PACT and JCSG+). First thin, plate-like crystals grew in the condition, containing PEG 4000 25% 0.1 M di-sodium citrate pH 5.6 and 0.2 M ammonium sulphate, within one or two weeks at 292 K (Figure 5b). The further biochemical characterization and optimization of crystallization conditions to produce crystals suitable for X-ray analysis is currently under progress.

## Discussion

Based on sequence analysis, a large proportion of genes from genomic data of marine organisms have unknown cellular and/or molecular functions. One major challenge is to assign biological function and to elucidate the mechanism of action of such genes. This challenge involves techniques to elucidate the structure and function of the gene products, interactions between proteins and/or global protein changes. For example, the three-dimensional structure of a protein can often provide functional clues, primarily by detecting structural similarity with a protein of known function even when sequence identity is low [9]. Purified protein is generally required in these studies and is at the basis of the development of medium/high throughput strategies to produce a large number of soluble proteins [10,11]. A key feature to the success of medium/high throughput cloning strategies is the optimization of an identical treatment of all targets. Moreover, multiplying constructs, vectors and targets consequently increase the chances to obtain pure, soluble protein samples to pursue biochemical analyses. This has also been demonstrated more recently by other expression systems, such as the ligation-independent cloning (LIC) method of *Mycobacterium tuberculosis *gene sequences [25].

The increased number of genomic projects concerning marine organisms that are available, including prokaryotic organisms [1,2] or eukaryotic organisms [3-7], as well as projects still in progress (*Ectocarpus siliculosus*, *Zobellia galactanivorans*), require the application of medium/high throughput transcriptomic and proteomic methods.

Here, we show that a general scheme for bacterial expression of genes originating from marine organisms could be successfully implemented for the production of soluble proteins. Relatively few studies have been performed to assess medium/high throughput expression of soluble proteins from marine organisms. However, in general efforts have been concentrated on individual organisms. For an example, the Southeast Collaboratory for Structural Genomics has developed high throughput protein production and crystallization of genes originating from *Pyrococcus furiosus *[26]. More recently, a series of diatom expression vectors based on the Invitrogen Gateway technology for high throughput protein tagging and overexpression in *Phaeodactylum tricornutum *has been described [27].

**Figure 6:**
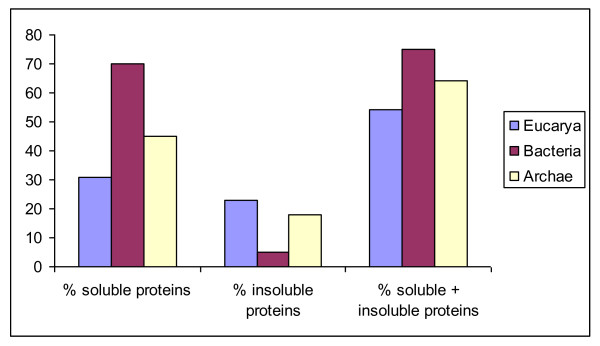
**Comparison of soluble and insoluble protein expression percentage in the three-domain system**. Global comparison of the successful expression obtained from archaea, bacteria and eukarya. Eukarya are represented by the blue bars, Bacteria are represented by the red bars and Archaea are represented by the yellow bars.

In the present study a medium throughput approach was applied for the heterologous protein expression of diverse marine organisms (including prokaryotes, archae and eukaryotes) in the same system (Figure 6). This is in contrast with previous studies that generally focus on one organism of origin. The general concept of the MARINE-EXPRESS program can be divided into three main parts: i) the bioinformatics analysis of the target genes selected by different partners, ii) the cloning of targeted genes into two different expression vectors and iii) the soluble expression of the targeted proteins. Table 1 shows the MARINE-EXPRESS consortium partners and the main organisms studied. Moreover, we have analyzed target genes from a lemon-shaped Virus-like Particle (PAV1) isolated from the hyperthermophilic euryachaeote *Pyrococcus abyssi *[28] and some target genes from the brown alga *Laminaria digitata *and the red alga *Chondrus crispus *[6,7]. To enhance protein solubility and to facilitate affinity chromatography in the next purification steps, we used a gene fusion approach. Several tags have been developed commercially to facilitate rapid single-step purification [29,30]. The most popular are glutathion-S-transferase (GST) [31] and polyhistidine (His_6_) [32] tags. The His_6_-tag is popular because of its small size. GST fusions help in obtaining soluble products of the whole fusion protein simply by its presence. Each expression scenario requires a specific vector. Re-cloning genes into each of these vectors is extremely labor-intensive. Recombinatorial cloning methods provide an opportunity to minimize the effort required for alternate expression [33]. The present study includes two expression vectors with either a His-tag (pFO4) or GST-tag (pGEX-4T-1) at the N-terminal that allow the same restriction-enzyme-based cloning in *Bam*HI/*Eco*RI. Figure 1 shows an average cloning success superior to 80%. This success rate is clearly higher than those obtained with different organisms [34] or at different centers from the Protein Structure Initiative [13]. The use of auto-induction media for small-scale protein production was successful. This is measured by the high level of target protein expression (>80%) that has been achieved.

The next stage, analysis of protein expression can be carried out without affinity purification using SDS-PAGE analysis which is a very reliable method, but not very sensitive. Indeed, for direct detection of the His-tagged product in the soluble fraction, a dot-blot procedure with an anti-His antibody is often applied [35-37]. Dot-blot is a fast method to screen expression and solubility of recombinant proteins using a convenient 96-well format. However, the reliability of this method is limited due to lack of specificity of the detection method, and it does not give information about the size and the purity of detected protein. One solution is to couple dot-blot with techniques providing information about the actual size such as capillary electrophoresis, SDS-PAGE or Western blotting. In out study, the use of affinity mini-columns increased the percentage of detected soluble targets by 15% for His-tagged and 60% for GST-tagged targets. Moreover, this method permitted to the expression level of recombinant proteins to be estimated more precisely and confirmed their correct molecular weight. In fact, the number of obtained soluble proteins is generally under evaluated. Indeed, we have seen that for some targets that were judged negative in small-scale experiments, culturing them in a larger volume of auto-inducible medium, such as 50 ml, in some cases allowed soluble expression of these 'negatively' judged targets (data not shown). The triage based on the small-scale results reduces the number of targets that progress to large-scale culture preparation [32], but in rare particular cases misses potential soluble expression.

Previous studies have indicated that approximately 50% of full-length proteins from the *Eubacteria *or *Archaea *and only 10-20% of proteins from *Eucarya *can be expressed in *E. coli *in soluble form [38,39]. This percentage has been significantly increased (nearly 50%) for human targets proteins using a multi-construct approach [40]. In the present study, 44% of soluble proteins were obtained. The best results are obtained for marine bacteria with 67% of soluble proteins, then archaea with 45% and as expected, *Eucarya *give the smallest percentage with 31%. These differences decrease if we take both insoluble and soluble proteins into account (Figure 6). In summary, we used a parallel production approach for bacterial expression in medium throughput to yield 84 soluble proteins from a total of 192 marine targets (44%).

While expression or crystallization strategies can be generalized to a common factor like their marine bacterial or eukaryotic origin, the setup for the functional screening is intimately bound to the gene family of interest to the different consortium members (Table 1). This latter step, performing the functional/biochemical characterization of the soluble expressed proteins, will therefore be conducted by each partner and will focus on the family of genes of their interest.

In conclusion, the present project provided purified proteins that are key reagents for numerous assays that address fundamental questions about their structure, function and regulation. For the first time, our medium throughput project allowed the expression of various proteins of marine origin in parallel, independent of organism. A rapid and cost-effective small-scale screening method for soluble expression of proteins from marine organisms in *E. coli *has been established, allowing the different partners to access large quantities of purified protein and to choose among targets of their interest for subsequent functional and/or structural analysis, which is currently underway.

## Methods

### Strains, plasmids and culture conditions

The sources and relevant genotypes of the bacterial strains as well as of the plasmids used are listed in Table 3. Plasmid pGEX-4T-1 (GE Healthcare Life Science, USA) is used to express protein in fusion with a glutathione-S-transferase (GST) at the N-terminal. Plasmid pFO4 (a vector modified from pET15b (Novagen, USA) to be compatible with the *Bam*HI/*Eco*RI ligation strategy) generates a hexa-histidine tail at the N-terminal of recombinant protein. *Escherichia coli *DH5α, used for standard cloning procedures, and *E. coli *strains used for gene expression experiments, were grown in Luria-Bertani (LB) liquid or on LB solid medium, as described by Sambrook *et al*. in 1989 [41]. For expression tests, an auto-inducible ZYP5052 medium [19] was used. All media were supplemented, when necessary, with 100 μg ml^-1 ^ampicillin (sodium salt).

**Table 3:** Escherichia coli strains and plasmids used in this study.

	*Genotypes and relevant properties*	*Sources or references*
***Strains***		

DH5α	F^- ^*end*A1 *gln*V44 *thi-1 rec*A1 *rel*A1 *gyr*A96 *deo*R *nup*G ϕ80d*lacZΔ*M15 Δ(*lacZYA-argF*)U169, *hsd*R17(rK- mK+), λ-	Promega, USA
BL21	F^- ^*ompT gal dcm lon hsdS_B_(r_B_^- ^m_B_^-^)*	Novagen, USA
BL21 (DE3)	F^- ^*ompT gal dcm lon hsdS_B_(r_B_^- ^m_B_^-^*) λ(DE3 [*lac*I *lac*UV5-T7 *gene *1 *ind*1 *sam7 nin5*])	Novagen, USA
Rosetta	F^- ^*ompT hsdS_B_(R_B_^- ^m_B_^-^) gal dcm*	Novagen, USA
Rosetta (DE3)	F^- ^*ompT hsdS_B_(R_B_^- ^m_B_^-^) gal dcm *λ(DE3 [*lac*I *lac*UV5-T7 *gene *1 *ind*1 *sam*7 *nin*5])	Novagen, USA
Origami	F^- ^*ompT hsdS*_B _r_B_^- ^m_B_^-^) *gal dcm lacY1 ahpC gor522*::Tn*10 *(Tc^R^) *trxB*::kan pAR5615 (Ap^R^)	Novagen, USA
Origami (DE3)	F^- ^*ompT hsdSB rB- mB-) gal dcm lacY1 ahpC gor522::Tn*10 (TcR) *trxB*::kan pAR5615 (Ap^R^) λ(DE3[*lac*I *lac*UV5-T7 *gene *1 *ind*1 *sam7 nin*5])	Novagen, USA

*Plasmids*		

pGEX-4T1	Amp^R^, tac promoter, GST.Tag	GE HealthCare, USA
**pFO4**	Amp^R^, T7lac promoter, His.Tag	This Study

### Bioinformatics analysis of the target sequences

The potential signal peptides and transmembrane domains have been predicted using SignalP and TMHMM, respectively [15,16]. The modularity of each target protein has been examined using Blast queries against UniProt database, as well as domain searches with the InterPro server [17]. The precise delineation of each module has been refined using Hydrophobic Cluster Analysis (HCA) [18]. For this study, 192 modules were chosen with predicted masses between 7 and 140 kDa and rearrayed into two 96-well plates. All procedures were performed where possible in this 96-well format.

### Primers design and cloning method

Expression vectors (pGEX-4T1 and pFO4) were digested by *Bam*HI and *Eco*RI. For each target sequence, we sought the restriction site recognized by *Bam*HI, *Eco*RI or their isocaudomers (respectively *Bgl*II and *Mfe*I) using BioEdit Sequence Alignment Editor (Ibis Biosciences Inc., USA). The target genes were classified into four compatible cloning strategies (*Bam*HI/*Eco*RI, *Bam*HI/*Mfe*I, *Bgl*II/*Eco*RI and *Bgl*II/*Mfe*I) in order to design the correct oligonucleotide primers, and assign targets in 96-wells plate). The standard scheme for primer design was defined as: for the forward primers, 5'-[hexa-*G *tail]-[*Bam*HI or *Bgl*II]-[Hybridization site]-3' and for the reverse primers, 5'-[hexa-*C *tail]-[*Eco*RI or *Mfe*I]-[*stop *anticodon]-[Hybridization site]-3'. Oligonucleotides for PCR were purchased in 96-well plates from Operon Biotechnologies GmbH (Cologne, Germany). PCR amplification was performed on a GeneAmp^R ^PCR System 2700 (Applied Biosystems, USA). The thermocycle utilized was: denaturation at 95°C for 5 min and thirty cycles of denaturing at 95°C for 30 s, annealing at 50°C for 30 s and polymerization at 72°C for 4 min. Template amplification was performed with Pfu polymerase (PROMEGA, USA) and used with the conditions recommended by the supplier. PCR reactions were analyzed on 1% agarose gels using standard procedures [41]. The resulting PCR products were purified using the QIAquick™ 96 PCR purification Kit (QIAGEN, USA), digested with appropriate restrictions enzymes and cloned in parallel into the pFO4 and pGEX-4T1 expression vectors using standard procedures [41]. PCR-screening was performed directly on the DH5α bacterial colonies to verify clones with inserts on expected size, using PCR primers which annealed upstream and downstream of the insertion site of pGEX-4T1 and pFO4. Target fragments were amplified using 10 μl of PCR Master Mix (PROMEGA, USA) added to 0.2 μl of each primer (100 μM) with the same program described above. Plasmid extraction was performed using MiniPrep SV purification Kit (PROMEGA, USA) and recombinant plasmids were used to transform *E. coli *expression strains.

### Screening for protein expression using 2 ml cultures

*E. coli *clones, for which the presence of the expression gene had been verified by colony PCR as described previously, were tested for the expression of the desired protein. Screening was done using 2 ml cultures in 24-deep well plates. Cultivation was performed in two phases. First, transformed colonies were grown at 37°C overnight in LB medium containing 100 μg ml^-1 ^ampicillin. Then, cultures were diluted 1:100 with auto-inductible ZYP5052 medium [19] containing 100 μg ml^-1 ^ampicillin and subjected to further incubation at 20°C until the desired density.

### Lysis of cells and detection of proteins

For solubility assay, cell pellets from small-scale expression cultures were resuspended in 500 μl of lysis buffer (Tris-HCl 50 mM, pH 7.5; NaCl 250 mM; EDTA 1 mM; lysosyme 1 mg ml^-1 ^; DNAse 0.1 mg ml^-1^) and incubated at 18°C for 1 hour and the soluble and insoluble fractions separated by centrifugation (12000 g, 20 min, 4°C). Insoluble pellets were resuspended in 200 μl of lysis buffer supplemented with urea 6 M. Samples from soluble and insoluble fractions were separated by 12% sodium dodecyl-sulfate polyacrylamide gel electrophoresis (SDS-PAGE) using 12% Criterion precast Bis-Tris gels with 26 wells. Targets were scored as positive for expression and solubility if a detectable fusion protein of the correct molecular weight was observed after Coomassie-staining. In parallel, soluble fractions were purified using His or GST Microspin columns (GE Healthcare Life Science, USA) according to the protocol recommended by the supplier. The results were also analyzed by 12% SDS-PAGE.

## Competing interests

The authors declare that they have no competing interests.

## Authors' contributions

GM, CB and MC have set up and designed the study; AG and CH have set up and performed all experiments; GM, AG, CH and AJ have performed bioinformatic analysis; AJ has performed cloning and expression experiments in round 2; ER, PP and IM have performed up-scale expression, purification and crystallization experiments of selected targets; DF, CG, DP, MB, HM, CB and GM have provided genomic material, as well as access to genomic data and designed follow up experiments. AG, CH and MC have analyzed the data and written the manuscript; all authors discussed the results and commented the manuscript and all authors read and approved the final manuscript.

## Supplementary Material

Additional file 1 **Table S1**. Summary of all 192 targets, listing the partners providing the targets, the organism of gene origin, the accession number (where known), the primers used for amplification and eventually the follow-up experiments that have been performed. Click here for file
